# Early histological advantage of CD271-positive adipose-derived mesenchymal stromal cells isolated from the infrapatellar fat pad in experimental knee osteoarthritis

**DOI:** 10.1186/s12891-026-10249-6

**Published:** 2026-07-21

**Authors:** Takumi Sakamoto, Nozomi Aoki, Takumi Shiotani, Makoto Kitade, Takamasa Noguchi, Syunsuke Saito, Hideaki Nakajima

**Affiliations:** Department of Orthopaedics and Rehabilitation Medicine, Faculty of Medical Sciences, University of Fukui, 23-3 Matsuoka Shimoaizuki, Eiheiji-cho, Yoshida-gun, Fukui, 910-1193 Japan

**Keywords:** Osteoarthritis, Mesenchymal stromal cells, CD271, Adipose-derived MSCs, Intra-articular injection

## Abstract

**Background:**

Mesenchymal stromal cells (MSCs) have been increasingly explored as a minimally invasive treatment for osteoarthritis (OA), primarily due to their anti-inflammatory, immunomodulatory, and analgesic properties. However, MSC populations are biologically heterogeneous, and optimal cell phenotypes for intra-articular therapy remain unclear. CD271 has been identified as a marker associated with MSC subpopulations with enhanced regenerative potential. The aim of this study is to evaluate whether intra-articular injection of phenotype-selected CD271-positive adipose-derived MSCs (AD-MSCs) provides additional structural benefits compared with unselected AD-MSCs in experimental OA.

**Methods:**

OA was induced in athymic rats by intra-articular injection of monoiodoacetate. Human infrapatellar fat pad-derived AD-MSCs were isolated and separated into CD271-positive fractions using magnetic-activated cell sorting. One week after OA induction, animals received intra-articular injection of saline, plastic-adherent AD-MSCs (PA-AD-MSCs), or CD271-positive AD-MSCs. Cartilage degeneration was assessed macroscopically and histologically using the Histologic/Histochemical Grading System (HHGS). Pain-related neuropeptides in dorsal root ganglia were analyzed by immunohistochemistry, and synovial inflammatory cytokines were quantified by ELISA.

**Results:**

Both MSC-treated groups showed significant attenuation of cartilage degeneration, reduced synovial TNF-α and IL-6 levels, and decreased expression of pain-related neuropeptides compared with controls. The CD271-positive AD-MSC group had significantly lower HHGS scores than the PA-AD-MSC group at day 14, whereas macroscopic cartilage scores, inflammatory cytokine levels, and pain-related neuropeptide expression were not significantly different between AD-MSC-treated groups.

**Conclusions:**

Intra-articular administration of AD-MSCs suppresses inflammation, pain-related signaling, and cartilage damage in OA. Enrichment for CD271-positive cells was associated with a modest early histological advantage, while anti-inflammatory and analgesic effects were comparable between AD-MSC-treated groups.

## Introduction

Osteoarthritis (OA) of the knee is one of the most common musculoskeletal disorders and a major cause of pain, functional impairment, and reduced quality of life, affecting more than 500 million people worldwide [1, 2]. Although its prevalence increases with age, obesity, and mechanical risk factors, OA is not limited to older individuals. OA is now recognized as a whole-joint disease involving not only articular cartilage but also the synovium, subchondral bone, menisci, ligaments, and infrapatellar fat pad (IPFP) [3, 4]. The IPFP and synovial membrane form a closely interconnected anatomo-functional unit within the knee joint and are increasingly recognized as active participants in OA pathophysiology rather than passive adipose tissue [5, 6]. Surgical interventions such as total knee arthroplasty and osteotomy are effective in advanced disease, but most patients are initially managed conservatively. However, current pharmacological and physical therapies primarily provide symptomatic relief and do not modify disease progression [2, 7]. Given the limited intrinsic repair capacity of articular cartilage, development of minimally invasive and disease-modifying treatments is a major unmet clinical need [1, 8].

Mesenchymal stromal cells (MSCs) have attracted attention as a potential therapeutic option for OA because of their multilineage differentiation capacity and paracrine effects [9–11]. Early studies focused on cartilage regeneration via scaffold-based implantation, but subsequent investigations showed that direct intra-articular administration of MSCs is technically simpler and clinically attractive [12, 13]. Accumulating evidence suggests that the therapeutic benefits of MSCs may be mediated predominantly through anti-inflammatory, immunomodulatory, and analgesic mechanisms, rather than direct cartilage regeneration [10, 14–16].

Adipose tissue is an advantageous source of MSCs due to its accessibility and high cellular yield compared with bone marrow [15, 17]. Experimental studies using processed lipoaspirate (PLA) cells have shown that intra-articular injection of adipose-derived cells suppresses synovial inflammation and pain-related behavior in OA models, although cartilage repair was limited, particularly in advanced disease [18, 19]. These findings suggest that specific MSC subpopulations may exert differential biological effects in the OA joint. Among the various adipose tissue sources, the IPFP has attracted particular interest because it is an intra-articular tissue that can be readily harvested during total knee arthroplasty. In addition to being an active component of the osteoarthritic joint, the IPFP has been identified as a promising source of AD-MSCs with superior chondrogenic potential compared with other adipose depots [20]. Accordingly, the present study utilized IPFP-derived AD-MSCs.

CD271 is a surface marker associated with a subpopulation of MSCs exhibiting enhanced regenerative and immunomodulatory properties [21–24]. CD271-positive MSCs show reduced angiogenic activity and improve early-stage cartilage and osteochondral repair compared with unselected MSCs [23]. However, the effects of intra-articular administration of CD271-positive adipose-derived MSCs (AD-MSCs) on inflammation, pain modulation, and cartilage degeneration in OA is insufficiently characterized. Therefore, the purpose of this study is to investigate the therapeutic effects of intra-articular injection of CD271-positive AD-MSCs compared with plastic-adherent AD-MSCs (PA-AD-MSCs) in a monoiodoacetate (MIA)-induced knee OA model. Cartilage degeneration, inflammatory responses, and pain-related molecular markers were examined to identify potential advantages of this defined AD-MSC subpopulation as a potentially relevant AD-MSC subpopulation for OA treatment.

## Materials and methods

### Experimental animals

All experimental procedures were performed in accordance with the ethical standards for pain research in conscious animals proposed by the International Association for the Study of Pain, as well as the ARRIVE guidelines for reporting animal experiments. The study protocol was reviewed and approved by the Animal Care and Ethics Committee of Fukui University, and all animal handling complied with institutional regulations. Female athymic rats (10 weeks of age; body weight 150–170 g) were purchased from CLEA Inc. (Tokyo, Japan), a licensed commercial laboratory animal supplier. Animals were housed two per cage and maintained under controlled environmental conditions (temperature 23 ± 1 °C; 12-hour light/dark cycle) with unrestricted access to standard chow and water throughout the experimental period.

### Isolation and culture of adipose tissue-derived mesenchymal stromal cells (AD-MSCs)

Human adipose tissue was obtained from the IPFP of donors undergoing total knee arthroplasty at our University Hospital (2 men and 4 women; age range, 62–74 years; Kellgren–Lawrence grade 3/4 [2/4 donors]; BMI, 29.9 ± 2.9 kg/m²) after written informed consent was obtained. Approximately 5–10 g of harvested adipose tissue was finely minced under sterile conditions and enzymatically digested using collagenase type I (0.075%; Sigma-Aldrich, St. Louis, MO, USA) at 37 °C for 1 h under gentle agitation. Following digestion, the cell suspension was passed through a 70-µm cell strainer and centrifuged to collect the stromal vascular fraction. The resulting cell pellet was washed three times with phosphate-buffered saline (PBS) and resuspended in high-glucose Dulbecco’s modified Eagle’s medium (DMEM; Gibco, Grand Island, NY, USA) supplemented with 10% fetal bovine serum. Cells were cultured under humidified conditions (5% CO₂, 37.5 °C) and expanded until reaching 80–90% confluence. Culture medium was replaced every 2–3 days, and cells were sub-cultured using trypsin–EDTA. Cells from passage III were used for all subsequent experiments.

### Magnetic-activated cell sorting for CD271-positive AD-MSCs

Magnetic-activated cell sorting was used to separate CD271-positive AD-MSC populations, as described previously [23]. AD-MSCs obtained from six donors were expanded separately to passage 3 and pooled prior to magnetic sorting to ensure sufficient cell numbers and reduce donor-to-donor variability. The pooled AD-MSCs were detached using trypsin–EDTA, washed, and resuspended in MACS buffer consisting of PBS supplemented with bovine serum albumin and EDTA. Cells were incubated with CD271-conjugated magnetic microbeads and Fc receptor blocking reagent at 4 °C for 20 min, allowing selective labeling of CD271-expressing cells. After incubation, cells were washed and applied to a magnetic separation column positioned in a magnetic field. Subsequently, the column was removed from the magnetic field and retained cells were eluted to obtain the CD271-positive population. CD271-positive cells were further cultured in high-glucose DMEM supplemented with 10% fetal bovine serum under humidified conditions (5% CO₂, 37.5 °C) and expanded to the required number for in vivo experiments.

### Flow cytometric characterization of AD-MSCs

Flow cytometry was performed to characterize surface antigen expression of cultured AD-MSCs. Single-cell suspensions were incubated with fluorochrome-conjugated antibodies against CD34, CD45, CD73, CD90, CD105, and CD271 (BD Biosciences, Franklin Lakes, NJ) (each 0.25 µg/100 µl) at 4 °C. Corresponding isotype-matched antibodies for each fluorochrome-conjugated antibody were used to define positive gates for each fluorophore. Data acquisition was performed using a FACSCanto II flow cytometer. At least 10,000 gated events were analyzed per sample. Antigen expression profiles were quantified using dedicated analysis software (FACSDiva; BD Biosciences) to confirm the mesenchymal phenotype of cultured cells and the absence of hematopoietic markers.

### Induction of osteoarthritis and retrograde neuronal labeling

Animals were anesthetized with inhalational isoflurane (2–3% in oxygen) and osteoarthritis was induced by intra-articular injection of MIA (1 mg in 25 µl of sterile 0.9% saline; final concentration, 40 mg/ml, Sigma-Aldrich) into the right knee joint. To identify sensory neurons innervating the knee, a retrograde fluorescent tracer (2% fluoro-gold (FG) in 25 µl of 0.9% saline; Fluorochrome, Denver, CO) was co-injected at the same site. Injections were performed through the infrapatellar ligament using a 27G needle with the knee flexed to ensure accurate intra-articular delivery. After recovery from anesthesia, animals were returned to their cages and monitored daily.

### Intra-articular administration of AD-MSCs

**Fig. 1:**
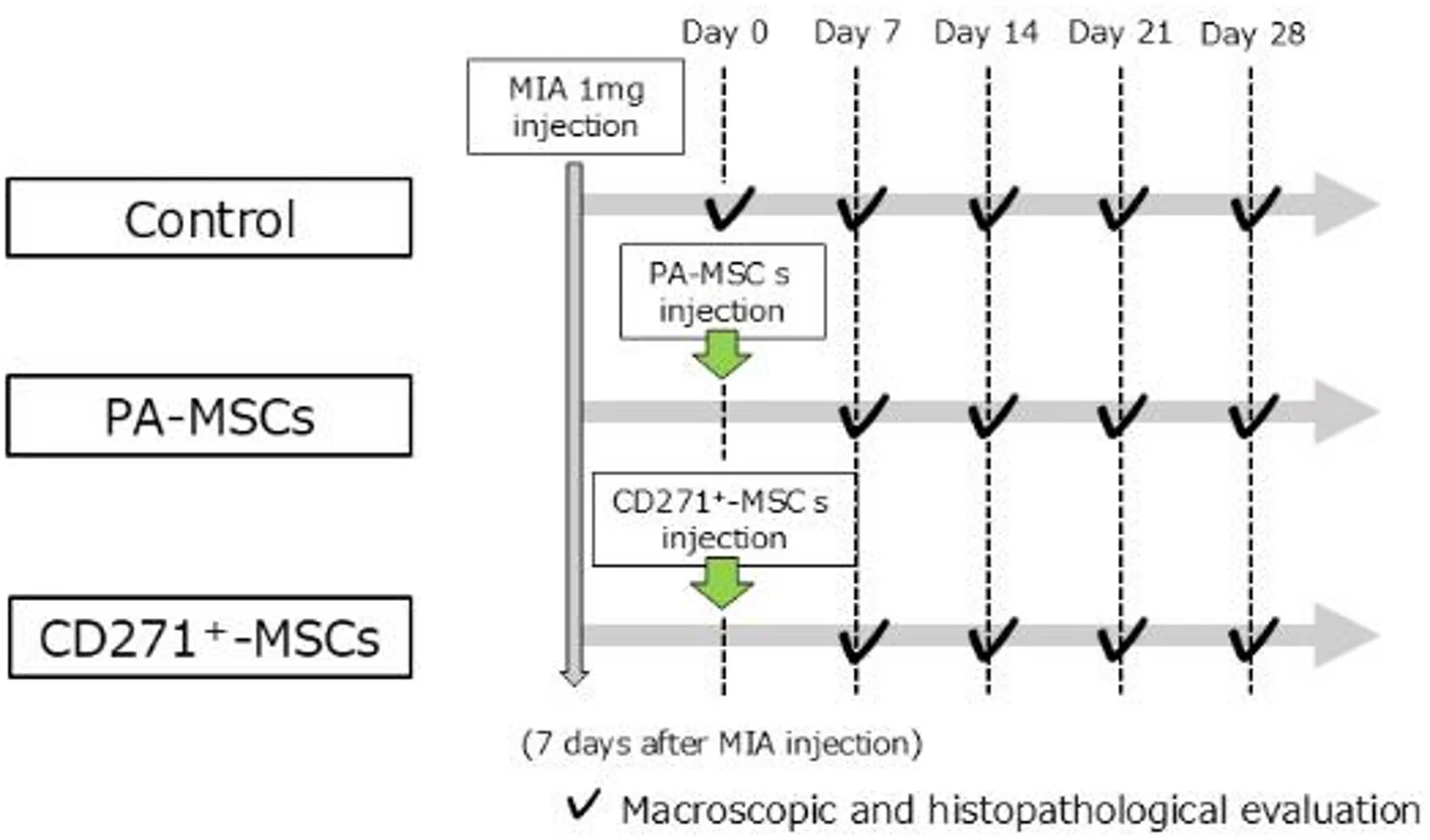
Experimental design and treatment protocol. Osteoarthritis (OA) was induced by intra-articular injection of monoiodoacetate (MIA). AD-MSCs were administered intra-articularly on 7 days after OA induction (Day 0). Animals were evaluated at days 7, 14, 21, and 28 after intra-articular injection

Animals were randomly assigned to one of three experimental groups: vehicle-treated controls (*N* = 30; *n* = 6 at baseline and each subsequent time point), unsorted plastic adherent AD-MSC (PA-AD-MSC)-treated (*N* = 24; *n* = 6 per time point), and CD271-positive AD-MSC-treated (*N* = 24; *n* = 6 per time point). On 7 days after osteoarthritis induction (Day 0), animals were re-anesthetized and intra-articular injection into the right knee of either 50 µl saline (vehicle control), 1 × 10⁶ unsorted PA-AD-MSCs at passage 3–5, or 1 × 10⁶ CD271-positive AD-MSCs at passage 3–5 suspended in 50 µl saline using a 27G needle. Animals were euthanized by an overdose of pentobarbital sodium at predetermined time points (7, 14, 21, and 28 days after intra-articular injection). Knee joints and spinal tissues harvested from the same indivisual animal were used for macroscopic evaluation, histopathological assessment, immunohistochemical analysis, and synovial cytokine measurement (Fig. 1).

### Tissue processing

Under deep anesthesia with isoflurane (4–5% in oxygen), animals were transcardially perfused with physiological 0.9% saline followed by 4% fixative solution after confirmation of loss of pedal withdrawal reflex. Animals were deeply anesthetized and transcardially perfused for tissue preservation. Death was confirmed by cessation of heartbeat and respiration before tissue collection. Knee joints, including surrounding synovial tissue and joint capsule, were carefully dissected for histological evaluation. Lumbar dorsal root ganglia (DRGs) at the L4 segment were harvested separately for immunohistochemical analyses. Joint specimens were fixed, decalcified, and processed for paraffin embedding, while neural tissues were cryoprotected and frozen for sectioning.

### Macroscopic and histological evaluation of the knee joint

Articular cartilage degeneration was assessed macroscopically using a standardized scoring system based on the extent of surface irregularity, erosion, and bone involvement [25]. The following criteria were used to assess the morphological changes of the femoral condyles. Grade 0: intact articular surface. Grade 1: ≤10 punctate depressions per condyle. Grade 2: >10 punctate depressions per condyle. Grade 3: Erosion of ≤ 50% of joint surface. Grade 4: Erosion of > 50% of joint surface. Grade 5: Bone destruction. The resected limbs were cut at the mid-femoral and mid-tibial levels and immersed in 4% buffered paraformaldehyde fixative for 1 week at 4 °C. The samples were then decalcified in KCX solution (Falma, Japan) for 24–48 h at 4 °C, dehydrated and embedded in paraffin, cut into 10-µm sections, and stained with hematoxylin and eosin and 0.1% safranin-O for 5 min for light microscopy. Cartilage degeneration was assessed using the Histologic/Histochemical Grading System (HHGS) [26], as a semi-quantitative evaluation of structural and compositional changes. Scoring was performed by two investigators blinded to group allocation to minimize observational bias, and the mean score was used for statistical analysis.

### Immunohistochemical analysis of dorsal root ganglia

DRG specimens were harvested and processed at 7, 14, 21 and 28 days after intra-articular injection. The specimens were treated overnight with 4% buffered paraformaldehyde fixative at 4 °C and then with PBS containing 20% sucrose for 20 h at 4 °C. They were then snap-frozen in liquid nitrogen before sectioning into 10-µm thick sections using a cryostat. Sections were incubated with rabbit anti-calcitonin gene-related peptide (CGRP) antibody (1:200, Abcam, Cambridge, UK) and goat anti-substance P (SP) antibody (1:200, Abcam) for 24 h at 4 °C, followed by incubation for 2 h at 4 °C with Alexa A11034 anti-rabbit IgG (to detect CGRP immunoreactivity) (1:500, Thermo Fisher Scientific, Waltham, MA, USA) or Alexa A11079 anti-goat IgG (to detect SP immunoreactivity) (1:500, Thermo Fisher Scientific). The sections were rinsed thrice in PBS after each step and the immunostained sections were assessed by fluorescence microscopy by an investigator who was blinded to the treatment. The number of FG-labeled DRG neurons with CGRP or SP immunoreactivity was counted using ImageJ software (National Institutes of Health, Bethesda, MD, USA) and calculated as a percentage of the total number of FG-labeled DRG neurons in each section.

### Measurement of synovial inflammatory cytokines

After euthanasia by transcardial perfusion under deep anesthesia, tissues were collected from the synovial membrane–joint capsule–IPFP complex under sterile conditions, with particular attention to the synovial membrane and adjacent joint capsule. The harvested tissues were homogenized in extraction buffer and centrifuged to eliminate insoluble material. The clarified supernatants were collected and subjected to enzyme-linked immunosorbent assay (ELISA) (RayBiotech, Peachtree Corners, GA, USA) for determination of tumor necrosis factor-α (TNF-α) and interleukin-6 (IL-6) concentrations. Cytokine levels were normalized to total protein content and expressed as pg/mg protein.

### Statistical analysis

All quantitative data are presented as mean ± standard deviation (SD). Because this study was designed as an exploratory preclinical experiment, sample size was determined with reference to previous comparable OA animal studies rather than by formal a priori power calculation. Normality of data distribution was assessed using the Shapiro–Wilk test. Ordinal variables, including macroscopic cartilage scores and HHGS, were analyzed using the Kruskal–Wallis test followed by appropriate post hoc multiple comparisons. Continuous variables, including cytokine concentrations and proportions of immunoreactive neurons, were analyzed using one-way analysis of variance (ANOVA) followed by Tukey’s multiple comparisons test. Comparisons were performed separately at each time point. A two-tailed p value < 0.05 was considered statistically significant. Statistical analyses were performed using EZR (Saitama Medical Center, Jichi Medical University, Saitama, Japan), a graphical user interface for R (The R Foundation for Statistical Computing, Vienna, Austria).

### Generative AI statement

The authors declare that Gen AI was used in creation of this manuscript. OpenAI’s ChatGPT (GPT-5.2) was used only for improving grammar, readability, and language of the text. After using this tool, the authors reviewed and edited the content as needed and take full responsibility for the content of the publication. No data analysis, interpretation, or scientific conclusions were generated by AI tools.

## Results

### Characterization of adipose-derived mesenchymal stem cells

**Fig. 2:**
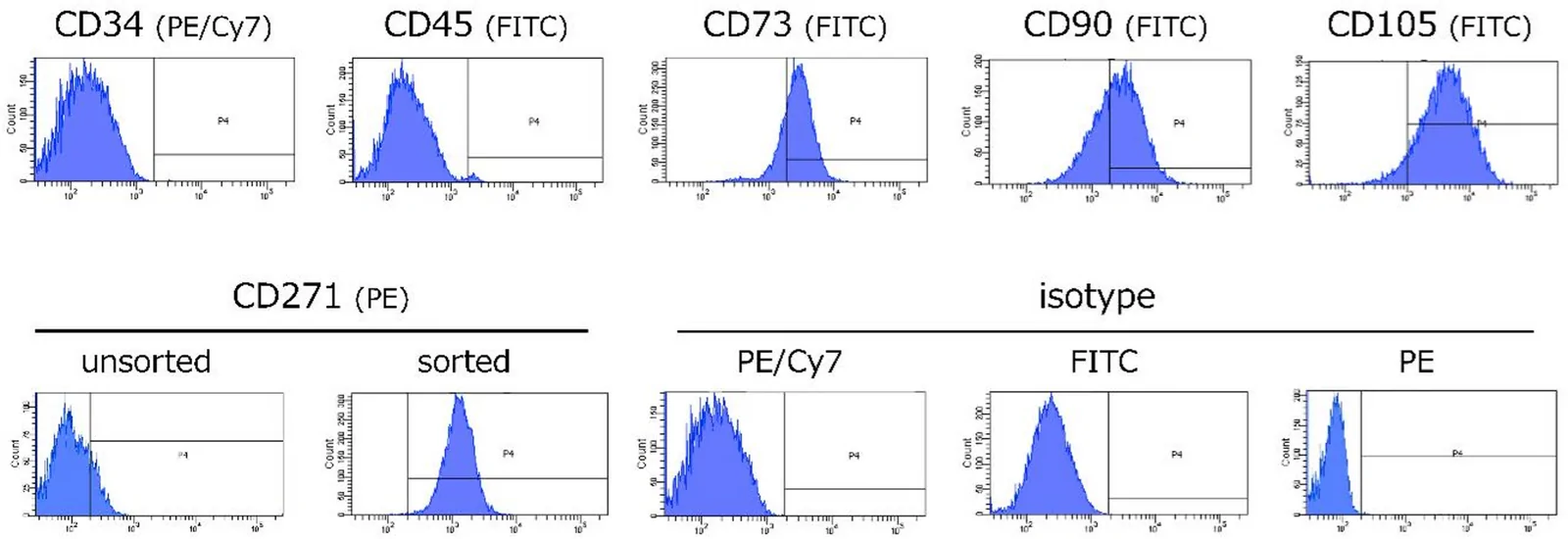
Representative flow cytometry histograms showing surface antigen expression of cultured AD-MSCs. Cells showed high expression of MSC markers CD73, CD90, and CD105, and minimal expression of hematopoietic markers CD34 and CD45, confirming their mesenchymal phenotype. CD271 expression was detected exclusively in the positively selected population, verifying successful magnetic-activated cell sorting

Cultured adipose-derived cells exhibited plastic adherence and spindle-shaped morphology. Flow cytometric analysis confirmed that the AD-MSCs expressed MSC markers CD73 (79.5 ± 6.6%), CD90 (64.5 ± 9.7%) and CD105 (92.9 ± 4.3%), with little expression of hematopoietic markers CD34 (0.1 ± 0.1%) and CD45 (1.2 ± 2.1%). The proportion of CD271-positive cells in unsorted PA-AD-MSCs was 19.4 ± 1.7%. Following magnetic-activated cell sorting, CD271-positive cells accounted for 99.8 ± 0.1% of the positively selected fraction, confirming successful magnetic separation (Fig. 2).

### Macroscopic evaluation of articular cartilage

**Fig. 3:**
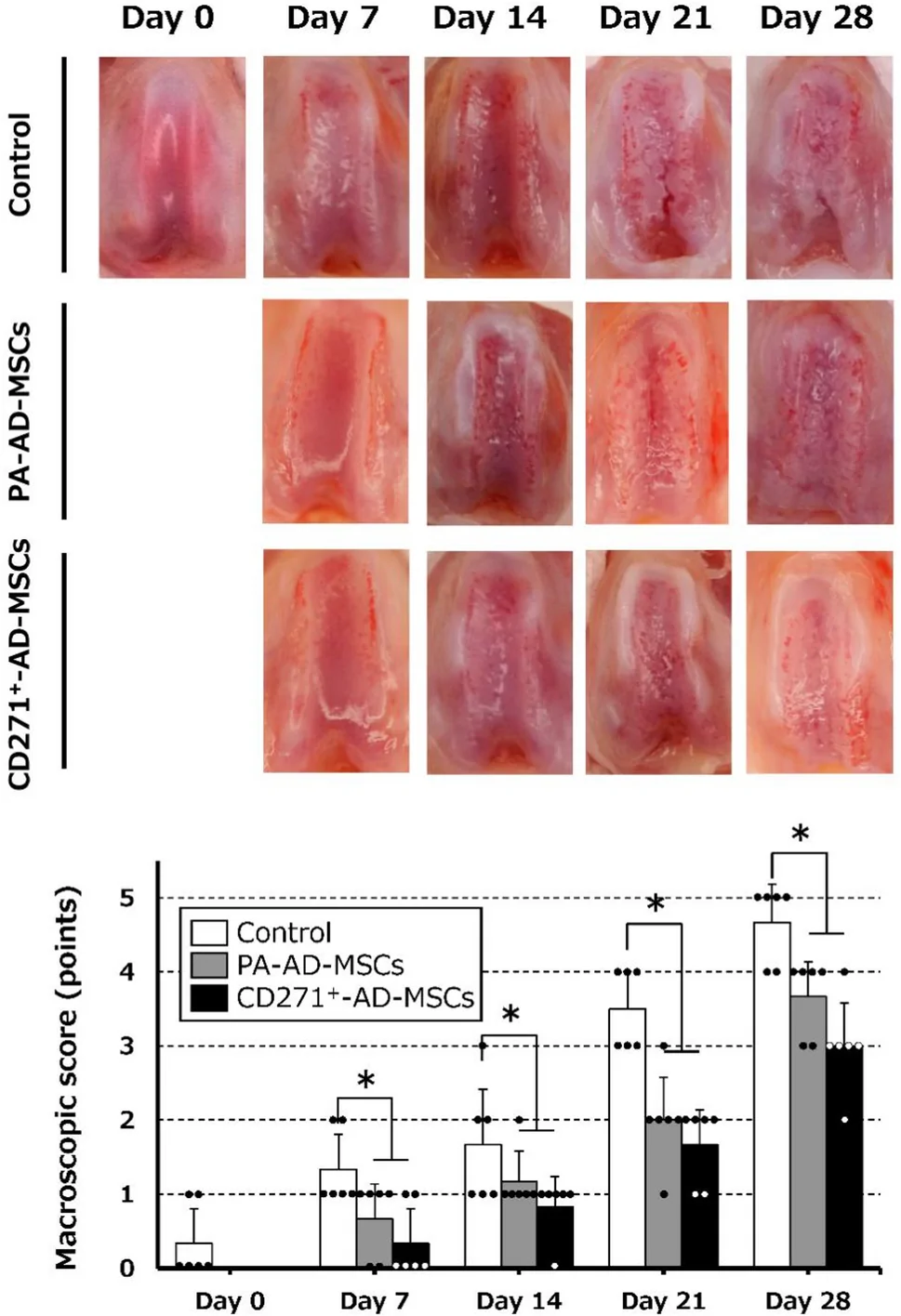
Macroscopic evaluation of cartilage degeneration after intra-articular AD-MSC treatment. Representative macroscopic images of femoral condyles at days 0, 7, 14, 21, and 28 after MIA-induced osteoarthritis. Progressive cartilage erosion was observed in controls, whereas PA-AD-MSC- and CD271⁺-AD-MSC-treated knees had relatively preserved articular surfaces. The AD-MSC-treated groups had significantly lower macroscopic scores for cartilage damage compared to controls from day 7 onward (*p* < 0.05). Differences between PA-AD-MSCs and CD271⁺-AD-MSCs were not significant. Data are shown as mean ± SD (*n* = 6 per group at each time point). *p < 0.05

Gross examination of the femoral condyles revealed progressive cartilage degeneration in the control group following MIA injection. In contrast, the PA-AD-MSC- and CD271-positive AD-MSC-treated groups both showed visibly preserved articular surfaces with reduced fibrillation and erosion compared with the control group throughout the observation period. Quantitative macroscopic scoring confirmed that cartilage degeneration was significantly attenuated in the AD-MSC-treated groups compared with controls. The CD271-positive AD-MSC group tended to show lower macroscopic scores than the PA-AD-MSC group at several time points, but with no statistical significance (Fig. 3).

### Histopathological assessment of cartilage degeneration

**Fig. 4:**
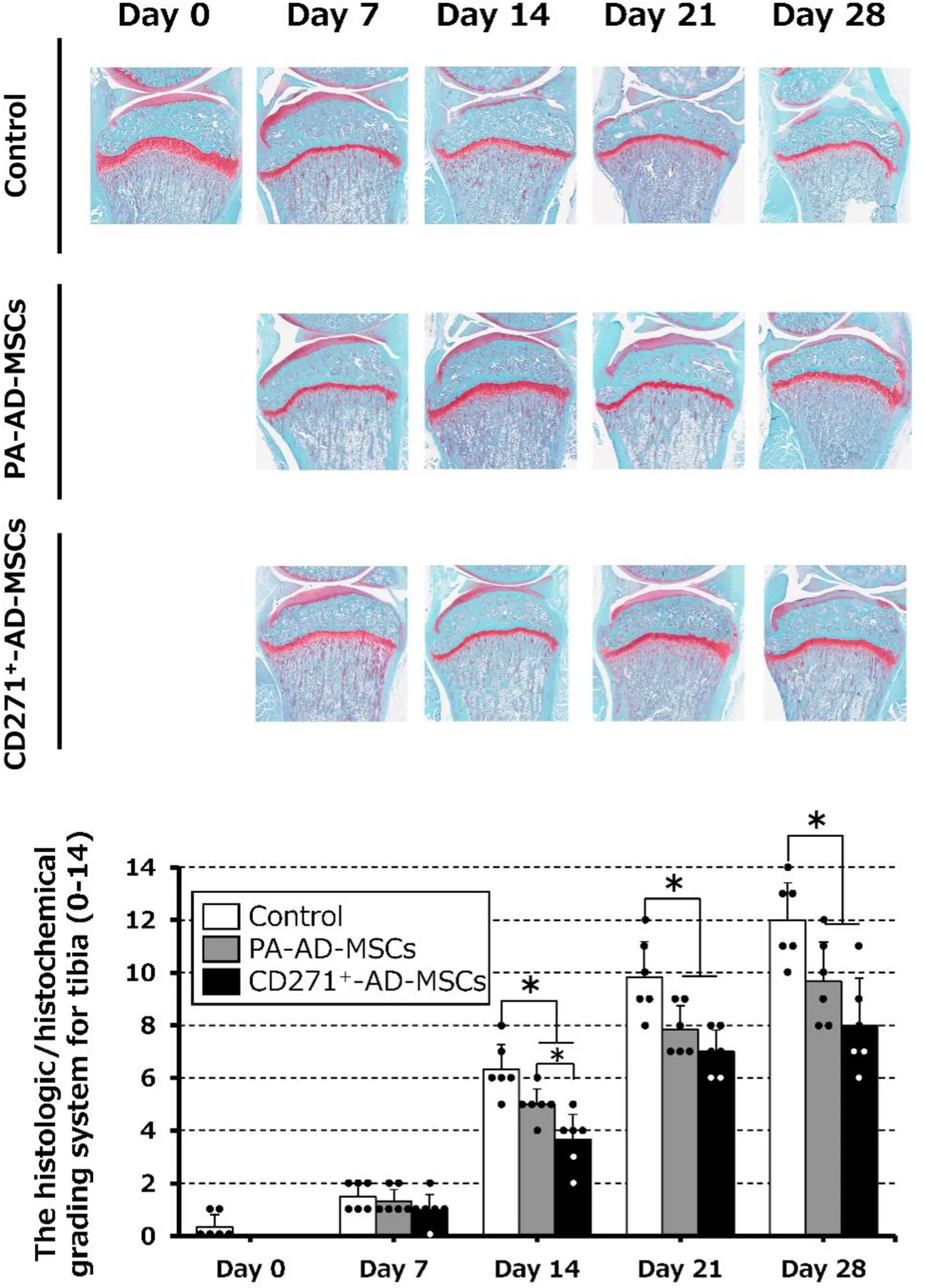
Histopathological evaluation of cartilage degeneration. Representative Safranin O–stained sections of knee joints at days 0, 7, 14, 21, and 28 after MIA-induced osteoarthritis. Progressive loss of proteoglycan staining and structural disruption of articular cartilage were observed in controls, whereas PA-AD-MSC– and CD271⁺-AD-MSC-treated knees had relatively preserved cartilage architecture and residual Safranin O staining. In quantitative histological assessment, both AD-MSC-treated groups had significantly lower HHGS scores than controls from day 14 onward (*p* < 0.05). The CD271⁺-AD-MSC group had significantly lower scores than the PA-AD-MSC group at day 14 (*p* < 0.05). Data are shown as mean ± SD (*n* = 6 per group at each time point). *p < 0.05

Safranin O and hematoxylin-eosin staining revealed progressive and marked loss of proteoglycan content together with structural disruption of articular cartilage in the control group over time. In contrast, the PA-AD-MSC- and CD271-positive AD-MSC-treated groups showed slower progression of cartilage degeneration, with residual Safranin O staining and relatively preserved cartilage architecture even at 28 days after intra-articular injection. Quantitative assessment showed significantly lower HHGS histological scores in both AD-MSC-treated groups compared to the control group from day 14 onward. The CD271-positive AD-MSC group had a significantly lower HHGS score than the PA-AD-MSC group at day 14 (Fig. 4). Although the CD271-positive AD-MSC group maintained lower HHGS scores at days 21 and 28, the differences between the two AD-MSC-treated groups were not statistically significant.

### Immunohistochemical analysis of pain-related neuropeptides in dorsal root ganglia

**Fig. 5:**
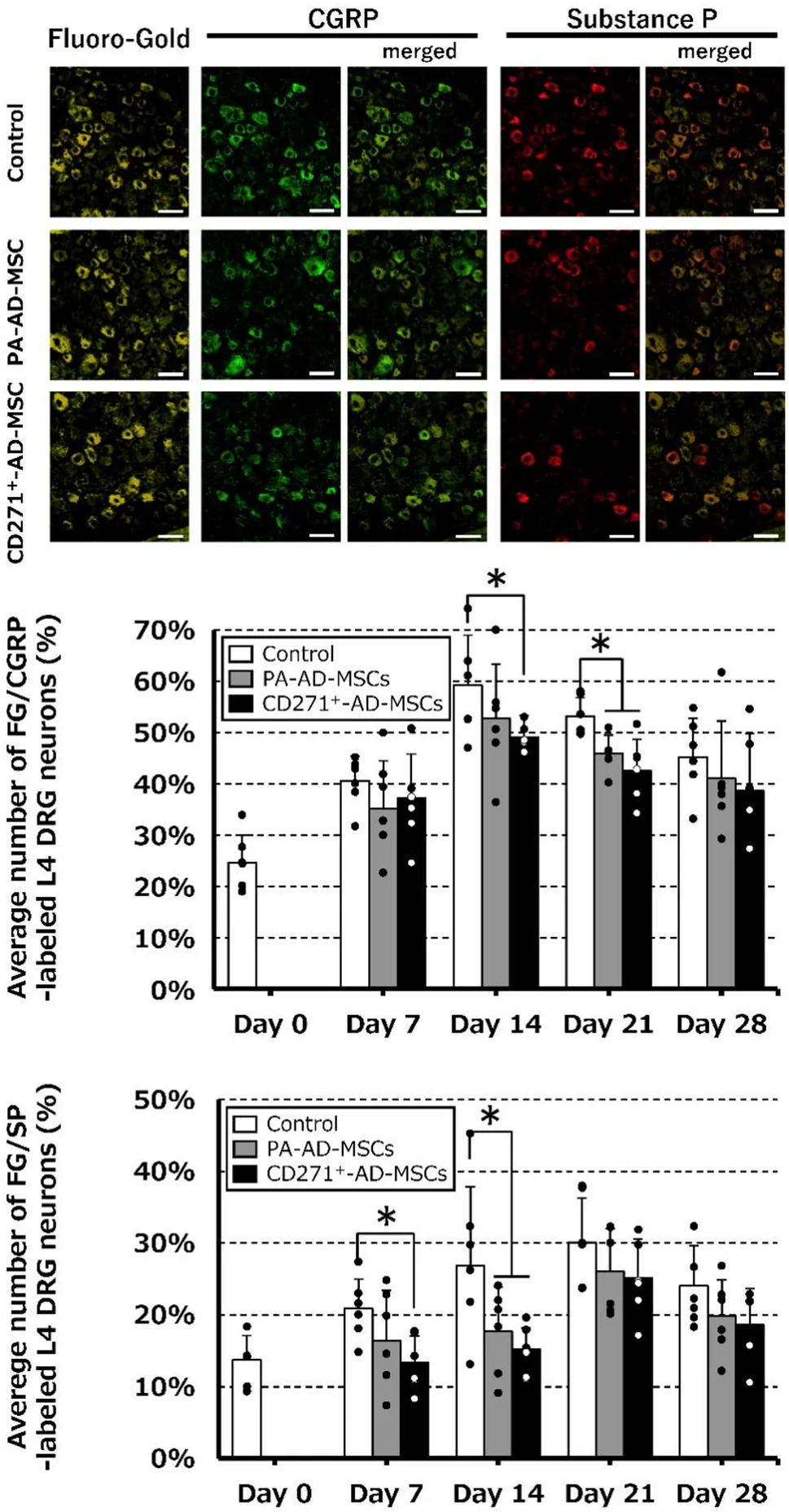
Immunohistochemical analysis of pain-related neuropeptides in dorsal root ganglia (DRGs). Representative fluorescence images at day 14 showing Fluoro-Gold (FG)-labeled DRG neurons innervating the knee joint and immunoreactivity for calcitonin gene-related peptide (CGRP) and substance P (SP). Merged images show co-localization of FG with CGRP or SP. Quantitative analysis revealed that the proportion of FG-labeled CGRP-positive neurons was significantly reduced in the CD271⁺-AD-MSC group at day 14 and in both AD-MSC-treated groups at day 21 (*p* < 0.05). For SP immunoreactivity, significant reductions were observed in the CD271⁺-AD-MSC group at day 7 and in both AD-MSC-treated groups at day 14 (*p* < 0.05). No significant differences between PA-AD-MSCs and CD271⁺-AD-MSCs were detected at any time point. Data are presented as mean ± SD (*n* = 6 per group at each time point). Scale bars = 100 μm. *p < 0.05

Retrograde labeling with FG identified DRG neurons innervating the knee joint. Immunohistochemical analysis showed that the proportions of CGRP- and SP–positive DRG neurons increased over time in the control group following induction of OA. In contrast, the CD271-positive AD-MSC- and PA-AD-MSC-treated groups tended to show suppression of these pain-related neuropeptides compared with the control group. Quantitative analysis revealed that the percentage of FG-labeled CGRP-positive neurons was significantly reduced in the CD271-positive AD-MSC group at day 14, and in both the CD271-positive AD-MSC and PA-AD-MSC groups at day 21. For SP immunoreactivity, significant reductions were observed in the CD271-positive AD-MSC group at day 7, and in the CD271-positive AD-MSC and PA-AD-MSC groups at day 14. No significant differences were detected between the CD271-positive AD-MSC and PA-AD-MSC groups at any time point (Fig. 5).

### Inflammatory cytokine levels in synovial tissue

**Fig. 6:**
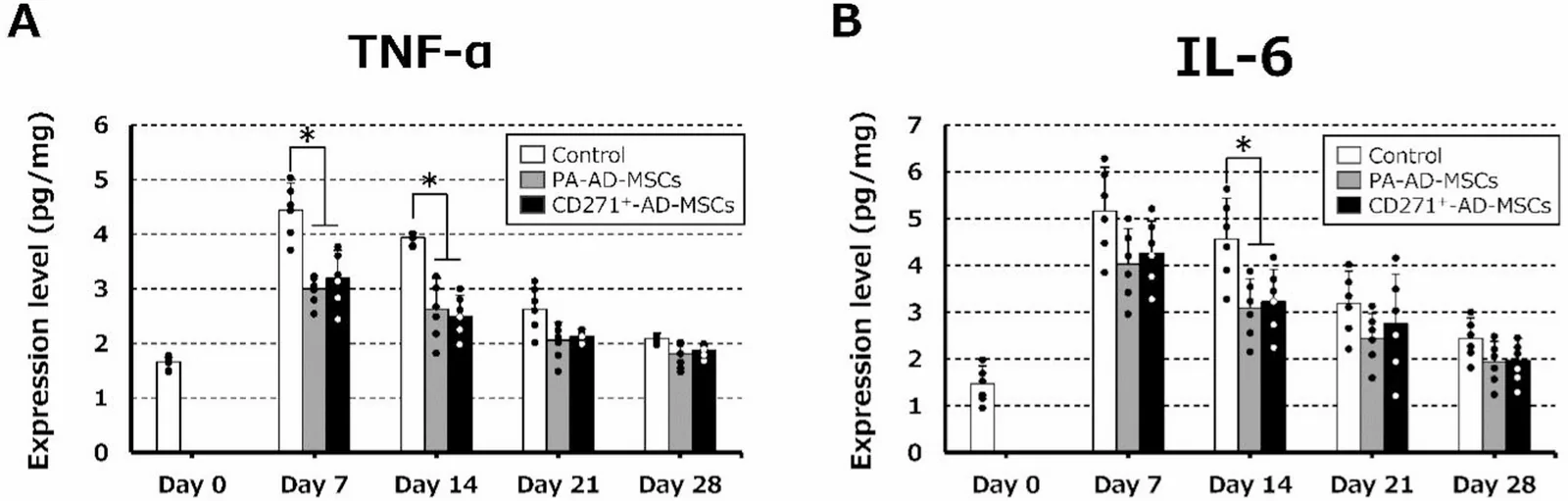
Periarticular inflammatory cytokine levels after AD-MSC treatment. Quantitative analysis of pro-inflammatory cytokines in periarticular tissue measured by ELISA. (**A**) TNF-α and (**B**) IL-6 at days 0, 7, 14, 21, and 28 after intra-articular injection. Both PA-AD-MSC- and CD271⁺-AD-MSC-treated groups had significantly reduced TNF-α levels compared with controls at days 7 and 14 (*p* < 0.05). IL-6 levels were significantly lower in both AD-MSC-treated groups at day 14 (*p* < 0.05). No significant differences were observed between PA-AD-MSCs and CD271⁺-AD-MSCs at any time point. Data are presented as mean ± SD (*n* = 6 per group at each time point). *p < 0.05

ELISA showed elevated levels of TNF-α and IL-6 in periarticular tissue following MIA-induced OA. Treatment with CD271-positive AD-MSCs and PA-AD-MSCs significantly reduced TNF-α levels compared with the control group at day 7 and 14 after intra-articular injection. Similarly, IL-6 levels were significantly lower in both AD-MSC-treated groups than in controls at day 14 (*p* < 0.05). No significant differences in TNF-α or IL-6 levels were observed between the CD271-positive AD-MSC and PA-AD-MSC groups at any time point. At days 21 and 28, no significant differences in TNF-α or IL-6 levels were observed among the groups(Fig. 6).

## Discussion

This study showed that both PA-AD-MSCs and CD271-positive AD-AD-MSCs attenuated cartilage degeneration compared with saline-treated controls. Compared with PA-AD-MSCs, CD271-positive AD-MSCs were associated with lower HHGS scores at day 14, whereas no significant between-group differences were observed in macroscopic scores, inflammatory cytokine levels, or pain-related neuropeptide expression. These findings suggest a limited early histological advantage of CD271-positive AD-MSC enrichment rather than broad therapeutic superiority. Because the between-group difference was limited to an early histological time point, the structural advantage of CD271-positive AD-MSCs should be interpreted as an early biological effect rather than definitive long-term superiority.

Over the past decade, the conceptual framework of MSC-based therapy for OA has undergone substantial revision. Initial enthusiasm was largely based on the assumption that transplanted MSCs would differentiate into chondrocytes and regenerate damaged cartilage. However, an increasing body of evidence suggests that durable engraftment of MSCs within the joint is limited and that their therapeutic actions are mediated predominantly through paracrine and immunomodulatory mechanisms [8, 9, 26]. Recent systematic reviews and randomized clinical trials have shown that intra-articular MSC administration can provide meaningful pain relief and functional improvement, even in the absence of clear structural cartilage restoration [14, 27–30]. The current findings are consistent with this modern perspective, demonstrating beneficial biological effects of MSC therapy while not implying complete cartilage regeneration.

Adipose tissue-derived MSCs are an attractive cell source due to their accessibility and high proliferative capacity [15, 17]. In the present study, all AD-MSCs were isolated from the IPFP, an intra-articular adipose tissue that has attracted increasing interest as both an active component of the osteoarthritic joint and a promising source of regenerative cells [5]. Although the IPFP contributes to OA pathophysiology through extensive interactions with the synovium, cartilage, and subchondral bone [5], previous studies have demonstrated that IPFP-derived MSCs possess enhanced chondrogenic potential compared with MSCs derived from other adipose depots [20]. However, because the IPFP is continuously exposed to the inflammatory microenvironment of the osteoarthritic joint, the biological characteristics of resident MSCs may also be influenced by disease-associated local signals. Previous studies have suggested that OA-IPFP-derived MSCs exhibit an inflammation-responsive phenotype with altered molecular and immunophenotypic characteristics while largely retaining their stem cell properties and regenerative potential [31]. Taken together, these characteristics support the use of the IPFP as a clinically accessible and biologically relevant source of AD-MSCs for OA research and regenerative therapy. Prior experimental work using heterogeneous adipose-derived cell preparations, including processed lipoaspirate cells, found significant anti-inflammatory and analgesic effects in OA models, but only modest structural improvement of cartilage [18, 19]. In agreement with these studies, the present results showed that unsorted PA-AD-MSCs can reduce inflammatory cytokines and DRG neuropeptide expression. Importantly, however, additional enrichment for CD271-positive cells resulted in lower HHGS scores at an early time point, may contribute to differences in early cartilage histology.

The rationale for focusing on CD271-positive MSCs is supported by recent studies showing that this subpopulation exhibits distinct biological characteristics compared with unselected MSCs. CD271 expression has been associated with enhanced clonogenicity and a more stable stromal phenotype [21, 23, 24]. Most prior reports have evaluated CD271-selected cells in the context of osteochondral defect repair. The present study extends these observations to a chemically induced OA model. Our data indicate that phenotype selection was associated with a modest early histological difference without altering the overall anti-inflammatory profile, as TNF-α and IL-6 suppression was similar with CD271-positive AD-MSCs and PA-AD-MSCs. This suggests that CD271 enrichment may preferentially influence mechanisms related to cartilage preservation rather than general immunomodulation.

Scaffold-assisted MSC delivery and hyaluronic acid (HA)–based carriers have been widely shown to improve cell retention and chondrogenic differentiation [12, 13, 32]. Experimental OA models have indicated partial structural repair when MSCs are combined with HA; however, these approaches increase procedural complexity and may limit clinical scalability [12, 33]. From a translational standpoint, minimizing the need for additional biomaterials may simplify manufacturing and regulatory processes, and thereby enhance clinical applicability.

Aberrant angiogenesis and sensory nerve ingrowth are increasingly recognized as central contributors to OA pain and structural progression [3, 4]. Modulation of these processes may be relevant, although these mechanisms were not directly examined in the present study. Emerging evidence also suggests that MSC-derived extracellular vesicles directly attenuate nociceptor sensitization and glial activation, contributing to pain relief independent of structural regeneration [34, 35]. These observations provide a plausible biological explanation for the analgesic effects observed in both MSC-treated groups and support the concept that MSC therapy exerts multifaceted actions on the osteoarthritic joint environment. An important observation was that the MSC-treated groups had reductions in CGRP- and SP-positive DRG neurons, reflecting attenuation of nociceptive signaling. These effects were observed in the absence of marked differences between CD271-positive AD-MSCs and PA-AD-MSCs, indicating that modulation of pain-related pathways may occur independently of the degree of structural cartilage protection. This dissociation is consistent with experiments showing that AD-MSC therapy can reduce OA-associated pain behavior through neuroimmune interactions [19, 34]. Therefore, our findings support the view that the modulation of pain-related molecular signaling of AD-MSCs are not solely dependent on cartilage regeneration, but may also reflect broader anti-inflammatory and neuromodulatory actions in the joint.

Synovial TNF-α and IL-6 levels were significantly reduced by both AD-MSC populations during the early post-treatment period, with no detectable difference between CD271-positive AD-MSCs and PA-AD-MSCs. These results suggest that suppression of major pro-inflammatory cytokines is a shared property of adipose-derived stromal cells and is not enhanced by CD271 selection. This observation aligns with reports indicating that AD-MSC-mediated immunomodulation involves multiple pathways and cell types, including macrophage polarization and modulation of synovial fibroblasts [12, 36]. The similar cytokine profiles in the two treatment groups imply that the additional chondroprotective effects of CD271-positive AD-MSCs may involve mechanisms other than generalized cytokine suppression.

Phase I/II clinical trials and meta-analyses of intra-articular MSC therapy for knee OA have shown favorable safety profiles and moderate symptomatic benefits, while indicating variability related to cell source, preparation, and dosing [21, 28–30, 37, 38]. Recent expert consensus statements advocate standardized biological characterization and potency assays for MSC products [39]. The present findings support this direction by showing that CD271-based phenotype selection was associated with limited histological differences in vivo. This emphasizes the importance of defined and reproducible cell populations for therapeutic optimization and for the establishment of reliable clinical protocols. In addition to phenotypic heterogeneity, the biological properties of MSCs may also be influenced by their tissue of origin. Because the IPFP is continuously exposed to the inflammatory milieu of the osteoarthritic joint, the biological properties of resident MSCs may be influenced by this local microenvironment, potentially affecting their immunomodulatory and reparative functions [5]. Although inflammatory priming was not directly investigated in the present study, tissue-specific conditioning of IPFP-derived MSCs may partly contribute to their therapeutic effects and represents an important topic for future investigation.

Several limitations should be acknowledged. First, the MIA-induced OA model was chosen for its reproducibility in evaluating early cartilage damage and inflammation, but it does not fully recapitulate the chronic biomechanical and degenerative features of human OA [6]. Second, the observation period was limited to 28 days, and longer-term effects of AD-MSC therapy were not evaluated. Third, although significant differences in cartilage preservation were detected, the molecular mechanisms underlying the additional histological effects of CD271-positive AD-MSCs were not investigated. Fourth, only the CD271-positive fraction was examined in vivo, because the non-retained fraction after magnetic sorting does not necessarily represent a pure CD271-negative population. Therefore, the present study cannot fully distinguish whether the observed structural effect reflects positive enrichment or depletion of other cell fractions. Fifth, pain-related outcomes were assessed by molecular markers in DRG rather than direct behavioral testing, and therefore functional analgesic effects cannot be determined from the present data alone. Finally, the use of human-derived cells in an immunodeficient rat model may not reproduce the biological responses expected in clinical settings. In addition, all AD-MSCs were isolated from osteoarthritic IPFP tissue, potentially reflecting biological conditioning by the chronic inflammatory joint microenvironment. Although isolation of CD271-positive AD-MSCs from rat adipose tissue was attempted, isolation of a distinct CD271-positive population was unsuccessful, necessitating the use of human-derived AD-MSCs. While the results show the potential advantages of CD271-based AD-MSC enrichment, further studies are required to validate these findings in additional OA models and to explore the durability of therapeutic effects. Comparative analyses incorporating detailed characterization of AD-MSC secretomes or extracellular vesicles may help to clarify the biological basis of phenotype-dependent differences. Nevertheless, the present data provide a rational foundation for considering cell phenotype as an important variable in optimization of AD-MSC therapy for OA.

## Conclusions

Intra-articular administration of infrapatellar fat pad-derived AD-MSCs attenuated cartilage degeneration, reduced synovial inflammatory cytokines, and suppressed pain-related neuropeptide expression in a rat model of knee OA. Compared with unsorted PA-AD-MSCs, CD271-positive AD-MSCs showed a lower HHGS score at day 14, while no significant between-group differences were observed in macroscopic cartilage scores, cytokine levels, or pain-related neuropeptide expression. These findings suggest a limited early histological advantage of CD271-positive enrichment and support the relevance of cell phenotype in AD-MSC-based approaches for OA.

## Data Availability

Data generated and analyzed during this study are included in this published article. Data and materials are available from the corresponding author subject to reasonable request and subject to the ethical approvals in place and materials transfer agreements.
